# *HACE1* deficiency causes an autosomal recessive neurodevelopmental syndrome

**DOI:** 10.1136/jmedgenet-2015-103344

**Published:** 2015-09-30

**Authors:** Ronja Hollstein, David A Parry, Lisa Nalbach, Clare V Logan, Tim M Strom, Verity L Hartill, Ian M Carr, Georg C Korenke, Sandeep Uppal, Mushtaq Ahmed, Thomas Wieland, Alexander F Markham, Christopher P Bennett, Gabriele Gillessen-Kaesbach, Eamonn G Sheridan, Frank J Kaiser, David T Bonthron

**Affiliations:** 1 Sektion für Funktionelle Genetik am Institut für Humangenetik, Universität zu Lübeck, Lübeck, Germany; 2 Section of Genetics, School of Medicine, University of Leeds, Leeds, UK; 3 Institute of Human Genetics, Technische Universität München, Munich, Germany; 4 Institute of Human Genetics, Helmholtz Zentrum München, Neuherberg, Germany; 5 Yorkshire Regional Genetics Service, Leeds, UK; 6 Zentrum für Kinder- und Jugendmedizin, Neuropädiatrie, Klinikum Oldenburg, Oldenburg, Germany; 7 Institut für Humangenetik, Universität zu Lübeck, Lübeck, Germany

**Keywords:** Genetics

## Abstract

**Background:**

The genetic aetiology of neurodevelopmental defects is extremely diverse, and the lack of distinctive phenotypic features means that genetic criteria are often required for accurate diagnostic classification. We aimed to identify the causative genetic lesions in two families in which eight affected individuals displayed variable learning disability, spasticity and abnormal gait.

**Methods:**

Autosomal recessive inheritance was suggested by consanguinity in one family and by sibling recurrences with normal parents in the second. Autozygosity mapping and exome sequencing, respectively, were used to identify the causative gene.

**Results:**

In both families, biallelic loss-of-function mutations in *HACE1* were identified. HACE1 is an E3 ubiquitin ligase that regulates the activity of cellular GTPases, including Rac1 and members of the Rab family. In the consanguineous family, a homozygous mutation p.R219* predicted a truncated protein entirely lacking its catalytic domain. In the other family, compound heterozygosity for nonsense mutation p.R748* and a 20-nt insertion interrupting the catalytic homologous to the E6-AP carboxyl terminus (HECT) domain was present; western blot analysis of patient cells revealed an absence of detectable HACE1 protein.

**Conclusion:**

*HACE1* mutations underlie a new autosomal recessive neurodevelopmental disorder. Previous studies have implicated *HACE1* as a tumour suppressor gene; however, since cancer predisposition was not observed either in homozygous or heterozygous mutation carriers, this concept may require re-evaluation.

## Introduction

Intellectual disability (ID) is characterised by the impairment of general mental abilities associated with defects in adaptive function. Many patients with ID exhibit additional clinical features or symptoms, such as motor abnormalities or epilepsy, reflecting variable developmental abnormalities of the brain. ID affects ∼2%–3% of the general population and, with recent advancements in genetic technology, there has been an increasing emphasis on identifying the genetic basis for neurodevelopmental disorders. The wide phenotypic spectrum of neurodevelopmental disorders reflects a huge diversity of underlying genetic and epigenetic pathologies.^1^ For single-gene inherited disorders, the pace of gene discovery has been greatly accelerated through the availability of whole exome sequencing. However, many neurodevelopmental disorders do not display phenotypic characteristics that are sufficiently distinctive to allow cohort ascertainment on a prospective basis. This has led to a variety of investigative approaches. It may be possible to map and identify causative genes within single large multiplex families, particularly if parental consanguinity can be exploited.^2^ Alternatively, heterogeneous cohorts of sporadic cases may be subjected to large-scale sequencing and post-hoc genetic classification, with varying degrees of success.^3^ In general, the pathogenicity of mutations identified within single families is likely to require additional support from unrelated cases or from other experimental sources. Searchable collaboration forums such as GeneMatcher^4^ may prove to be increasingly valuable in this respect.

Here, we describe two families in which eight individuals displayed a variable neurodevelopmental phenotype with ID, spasticity and abnormal gait. Because of the consanguinity in one family and sibling recurrences in the other, an autosomal recessive inheritance was suspected. Autozygosity mapping followed by candidate gene sequencing and exome sequencing were used to identify loss of function mutations in *HACE1*, which encodes a HECT domain- and ankyrin repeat-containing E3 ubiquitin protein ligase. HACE1 is believed to regulate the activity of a number of small GTPases, and previous genetic studies have mostly implicated it as a tumour suppressor.

## Methods

### Patients

A consultant clinical geneticist evaluated all patients at least once and comprehensive clinical history, examination and review of medical notes were performed and documented. Longitudinal information over a number of years was available for most of the individuals. DNA samples were obtained after written informed consent from subjects or their parents, according to a protocol approved by the Research Ethics Committees in Leeds (East) (reference 07/H1306/113) and the University of Lübeck.

### Gene identification

**Figure 1:**
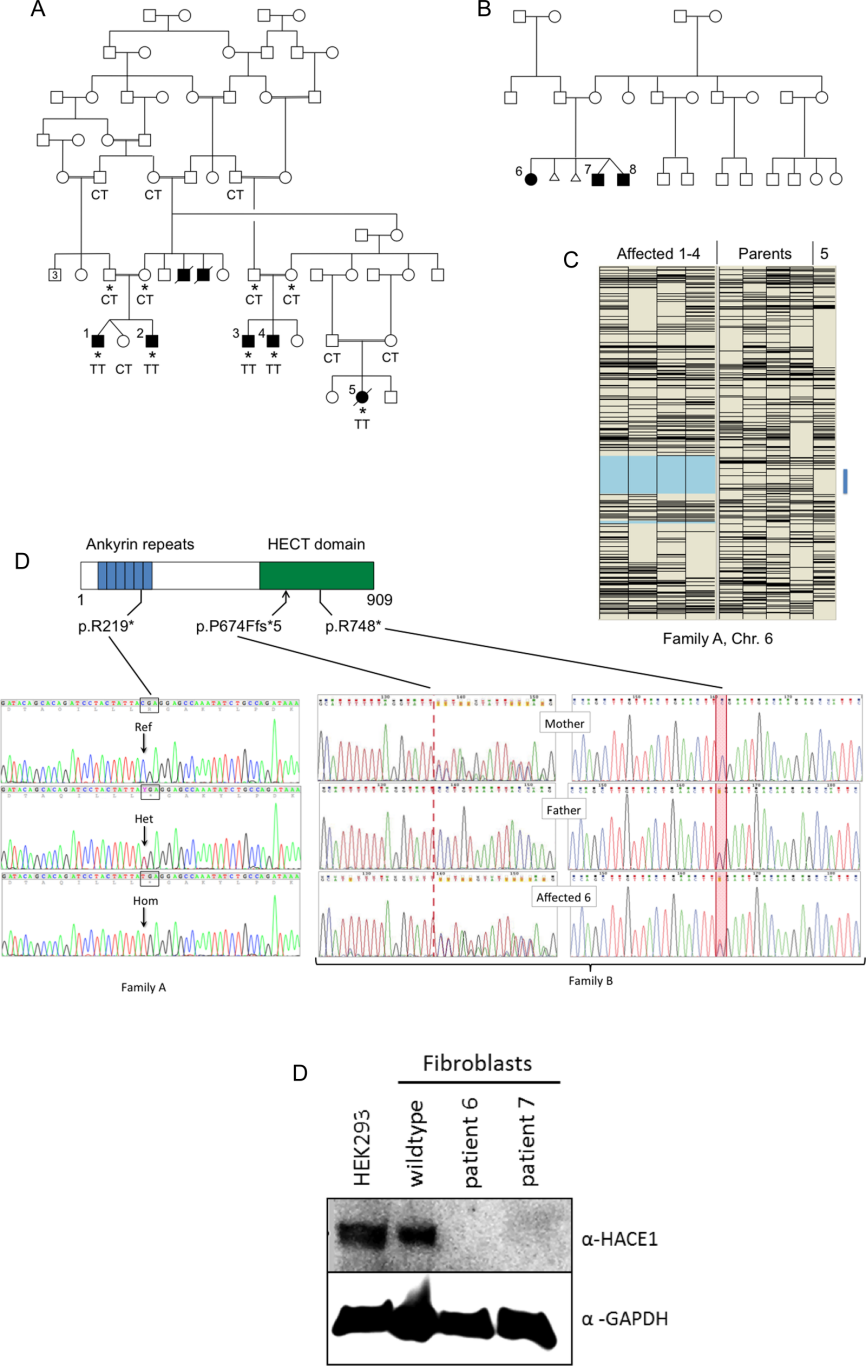
(A) Simplified pedigree of family A. Affected individuals are shaded and numbered 1–5. The two deceased males with shaded symbols were reported to have been affected with clinical features similar to those of patients 1–4. Asterisks indicate individuals used for autozygosity mapping (C). Genotypes at position c.655 (reference sequence C, encoding arginine 219; mutant allele T) are shown. (B) Pedigree of family B. (C) Autozygosity mapping using the ‘heterozygous SNPs’ view of AutoSNPa.^5^ Heterozygous SNPs (Affymetrix GeneChip Mapping 10K Array) appear as black horizontal bars. Patients 1–4 have been assigned as affected, resulting in delineation of a 24 Mbp region of Chr.6 (pale blue shading) that was concordantly homozygous. Patient 5 also has an interval devoid of heterozygous SNPs within the same region (blue bar); the genotypes are again concordant in this subject (not shown). (D) Position of the truncating mutations within the domain structure of HACE1 and illustrative Sanger sequence electropherograms of genomic PCR amplicons. In family B, the segregation of the compound heterozygous mutations is shown. (E) Western blot analysis for HACE1 of cultured fibroblasts from patients 6 and 7.

Genotyping of family A (affected individuals and parents, see figure 1A) was initially performed using the Affymetrix GeneChip Mapping 10K array; patients 1–5 were also later genotyped using the genome-wide human SNP 6.0 array, permitting finer resolution (see online supplementary figure S1). Data were analysed for autozygosity using AutoSNPa;^5^ for mapping purposes, only patients 1–4 were grouped as affected, because of the phenotypic differences seen in patient 5. A single large region of concordant homozygosity, encompassing a genomic interval of ∼24 Mbp, was shared by these four affected individuals (figure 1C).

To enrich the regions of interest prior to sequencing, we used the SureSelect in-solution method (Agilent). Biotinylated oligonucleotide baits were designed by extracting all coding regions from the University of California, Santa Cruz (UCSC) genome browser for the 993 unique RefSeq genes in the 24 Mbp Chr.6 locus, comprising a total target of 181 683 bp. These regions were uploaded to Agilent's ‘eArray’ software for automated oligonucleotide synthesis (in parallel with regions for seven other unrelated disease loci^6^). All subsequent steps, including genomic DNA preparation, target enrichment, library preparation, sequencing on an Illumina GA-II, read alignment and variant analysis, were performed as described.^6^ Mean depth of coverage for targeted regions was 81, with 96% of target bases covered by at least five reads of sufficient base quality for variant calling (Phred quality scores ≥17, mapping quality ≥20).

Exomes were enriched in solution with SureSelect XT Human All Exon 50 Mb kits (V.3, Agilent). Sequencing was performed as 100 bp paired-end runs on a HiSeq2000 system (Illumina) generating 8–10 Gbp of sequence with an average read depth of 100 and 92% of the target regions covered at least 20 times. Sequence analysis was performed as described.^7^

### Mutation verification

Sanger sequencing of patients and other family members was performed after PCR of genomic DNA derived from whole blood. Sequencing was performed using BigDye Terminator V.1.1 on an ABI 3730 (Applied Biosystems, Carlsbad, California, USA). Primer sequences and PCR conditions are available on request. Nucleotide numbering of *HACE1* variants is relative to reference sequence NM_020771.3.

### Cell culture and immunoblotting

Primary dermal fibroblasts of patients 6 and 7 (of family B) were cultured in Dulbecco’s modified Eagle’s medium (PAA Laboratories, Austria) supplemented with 10% foetal bovine serum and 1% penicillin–streptomycin at 37°C in a saturated humidified atmosphere containing 5% CO_2_. Cells were lysed in an adequate volume of radioimmunoprecipitation assay (RIPA) buffer. Protein concentration was determined using the colorimetric Bradford assay, separated by sodium-dodecyl-sulfate-polyacrylamide gel electrophoresis, and transferred to a polyvinylidene fluoride (PVDF) membrane (Roche, Mannheim). HACE1 was visualised by the use of an anti-HACE1 antibody (ab133637, Abcam) and an anti-GAPDH antibody (Cell Signaling) was used as a loading control.

## Results

### Clinical features

In *family A*, originating from the Mirpur region of North-Eastern Pakistan and Kashmir (figure 1A), five individuals had an undiagnosed neurodevelopmental disorder. The affected subjects all had severe learning difficulties. Their parents all had a normal phenotype.

In all affected individuals, hypotonia was noted either at birth or by 3–4 months of age, and there were delayed early motor milestones. All subsequently developed slowly progressive bilateral lower limb spasticity. Lower limb power and function were poor and most affected individuals were wheelchair users or bed-bound, though two were mobile over short distances with support. There was no evidence of wasting or fasciculation in the lower limbs, and sensation was preserved. Epilepsy developed in all of the patients early in childhood. Language acquisition was confined to a few single words and understanding was limited. All were doubly incontinent.

Three of the four male subjects had hypoplastic genitalia noted at birth. All four also had large heads from birth (91st–99th centile). All had problems with weight gain and most were significantly overweight.

Patient 5 had additional problems. She developed presumed viral encephalitis at age 1.5 years, requiring mechanical respiratory support for 6 days; this episode seemed to cause significant neurological regression. (Nonetheless, hypotonia and developmental delay had been noted prior to this.) Her occipitofrontal head circumference at birth had been on the 75th centile, but was on the 2nd centile by age 3. She died aged 9 years secondary to a chest infection.

All five patients had ocular abnormalities: myopia, divergent strabismus and/or retinal dystrophy. Sensorineural hearing loss was diagnosed in three of the five patients.

Family B (figure 1B) was of German descent. A sister and two dizygotic twin brothers all presented with a similar clinical syndrome comprising cognitive impairment, a hypotonic/ataxic movement disorder resulting in an unsteady, broad-based gait (see online supplementary video S1) and facial muscular hypotonia with inarticulate speech. Patient 6 displayed developmental delay. She was able to walk alone at the age of 4 years. Speech development was severely delayed. She exhibits a lumbar lordosis and a hypotonic-ataxic, intermittently dystonic movement disorder. Patients 7 and 8 are the younger twin brothers of patient 6. Patient 7 also presented developmental delay; independent walking started at 3–4 years. Speech started at a normal age but he has significant articulation problems. He developed epilepsy at the age of 2 years which is treated with antiepileptic drugs. He manifests the hypotonic-ataxic movement disorder most severely of the three siblings. His twin brother has the mildest phenotype of the sibship. Independent walking was achieved at 4½ years. In addition to a waddling gait, he shows bilateral lower limb spasticity.

Genetic investigations for subtelomeric rearrangements, for Prader–Willi and Rett syndromes and for myotonic dystrophies types 1 and 2 proximal myotonic myopathy (PROMM) were all normal.

The clinical features of the eight affected individuals are summarised in table 1, while photographs are shown in online supplementary figure S2.

**Table 1:** Clinical features of eight affected individuals from families A and B

Patient	Family A					Family B		
	1 (male)	2 (male)	3 (male)	4 (male)	5 (female)	6 (female)	7 (male)	8 (male)
Gestation at birth (weeks)	38	40	40	40	41	39	40	40
Birth weight (kg)	2.26 (2nd centile)	3.3 (25th centile)	3.85 (50th–75th centile)	3.74 (50th–75th centile)	4.05 (91st centile)	3.37 (mean)	3.4 (−0.9 SD)	3.2 (−1.5 SD)
Birth OFC (cm)	36.0 (91st–98th centile)	36.4 (75th–91st centile)	38.2 (98th–99th centile)	38.4 (98th–99th centile)	35.4 (75th–91st centile)	33 (−1.2 SD)	34 (−1.8 SD)	34.5 (−1.1 SD)
Hypogenitalism	Yes	No	Yes	Yes	No	No	No	No
Lower limbs	Bilateral spasticity	Bilateral spasticity	Bilateral spasticity	Bilateral spasticity	Bilateral spasticity	–	–	Bilateral spasticity
Upper limbs	Normal examination	Dystonic posturing	Normal examination	Normal examination	Increased tone and hyperreflexia	Normal	Dystonic posturing	Normal
Epilepsy	Myoclonic and tonic–clonic epilepsy from 7 months	Myoclonic and tonic–clonic seizures from 5 years	None	Tonic–clonic epilepsy from 10 years	Myoclonic epilepsy from infancy	None	Myoclonic seizures, focal epilepsy	None
Recent growth parameters								
Age	18 years	15 years	22 years	19 years	3 years	10 years 11 months	7 years 8 months	7 years 8 months
BMI (kg/m^2^)	33.3	37	55.7	50.4	20.6	24.8	21.17	19.32
OFC (cm)	59 (75th–91st centile)	59 (91st–98th centile)	64 (>99th centile)	63 (>99th centile)	47.2 (<0.4th centile)	54 (+0.5 SD)	53 (+0.2 SD)	53 (+0.2 SD)
Mobility	Never walked	Never walked	Age 16: mobile for short distances with callipers	Age 15: 10–15 steps with a rollator	Never walked	Unstable, waddling gait	Unstable, waddling gait	Unstable, waddling gait
Ophthalmic findings	Divergent strabismus	Divergent strabismus	Right divergent strabismus, bilateral myopia, right macular hypoplasia, retinal dystrophy	Divergent strabismus, myopia, bilateral retinal dystrophy	Severe myopia, left divergent squint, sluggish pupil responses, retinal dystrophy			Convergent strabismus, bilateral myopia, retinal dystrophy
Hearing	Bilateral sensorineural loss (40 dB)	No abnormality	Bilateral sensorineural loss (30 dB)	No abnormality	Bilateral sensorineural loss (40 dB)	No abnormality	No abnormality	No abnormality
Neurological investigations	*CT scan*: cerebral underdevelopment and marked atrophy of frontal and temporal lobes. *EEG*: consistent with myoclonic epilepsy	No cranial imaging performed	*CT scan*: generalised cerebral atrophy	*EEG*: normal *CT scan*: ventricular dilatation	*EEG*: focal and generalised spike-like discharges. Nerve conduction studies normal. *MRI brain*: prominent generalised cerebral and brain stem atrophy, disproportion between grey and white matters. *ERG*: Consistent with cone-rod dystrophy	*MRI*: hypoplastic corpus callosum *EEG*: normal	*MRI*: hypoplastic corpus callosum *EEG*: consistent with myoclonic epilepsy	*MRI*: enlarged ventricles *EEG*: normal
Skeletal		Left talipes equinovarus Kyphosis	Erb's palsy Right subtalar fusion age 9 years	Calcaneovalgus deformity of feet	Kyphoscoliosis Bilateral hip dislocation		Pes planus	Mild talipes equinovarus

BMI, body mass index; ERG, electroretinography; OFC, occipitofrontal head circumference.

### Identification of HACE1 as the causative gene

The complex consanguinity in family A allowed autozygosity mapping, while in family B, the affected individuals were analysed by exome sequencing.

Patient 5 was set aside in the initial autozygosity mapping, because of her phenotypic differences and consequent uncertainty about whether she had the same disorder as patients 1–4. The latter were found to share a single 24 Mbp concordant autozygous region on Chr.6q (figure 1C). Patient 5 was genotyped in parallel, however, and noted to have a region of concordant homozygosity within the same region of Chr.6q, bounded by heterozygous SNPs rs2388039 and rs2016207 (chr6:97 747 244–109 982 160 on the hg19 build). Inclusion of patient 5 and SNP 6.0 data obtained subsequently (see online supplementary figure S1) would allow narrowing the common concordant autozygous region to a 9.9 Mbp region between rs158777 and rs6903501 (98 959 605–108 848 121).

We sequenced all the genes within the larger 24 Mbp autozygous interval after enrichment by solution hybridisation, identifying a total of 259 sequence variants. Of these, 44 remained after filtering out SNPs present in dbSNP129 with a minor allele frequency (MAF) >0.01.

Only two of these variants were functional. A missense change in *MDN1* (NM_014611: c.4258A>G, p.M1420V) was predicted to be tolerated. A homozygous nonsense mutation c.655C>T was identified within exon 8 of *HACE1*. Within the predicted HACE1 protein structure, this mutation (p.R219*) lies within the fifth of the six N-terminal ankyrin repeats (figure 1D); the truncated mutant protein is thus lacking the entire catalytic HECT domain. Patients 1–5 were all homozygous for this mutation.

In family B, DNA from the three affected individuals 6–8 was subjected to exome sequencing. We used ∼6000 in-house exomes from individuals with unrelated diseases to filter rare variants (see online supplementary table S1). Using a MAF threshold of 0.02, we detected only a single gene, *HACE1*, with homozygous or compound heterozygous variants common to all three siblings. In *HACE1*, we found the mutations c.2242C>T and c.2019_2020insTTTAGGTATTTTTAGGTATT. The first of these predicts the protein truncating change p.R748* and was shown by Sanger sequencing to be paternally derived. The 20 nt insertion c.2019_2020insTTTAGGTATTTTTAGGTATT was derived from the mother. The last 12 nt of this insertion are a duplication of the reference genomic sequence immediately preceding the insertion point. If translated, this frameshifting mutation predicts the almost immediate truncation of the protein p.P674Ffs*5.

Homozygous or compound heterozygous occurrences of rare non-synonymous *HACE1* variants were only infrequently observed in control individuals. Our in-house exomes contained only one further exome carrying a homozygous *HACE1* missense variant, NM_020771.3:c.2659A>3, p.Ser887Arg. (This was in an individual diagnosed with macrocephaly that was most likely caused by a homozygous frameshift mutation in TBC1D7.) In addition, only three rare (MAF<0.2) homozygous non-synonymous missense variants (rs146393808, rs137941861, rs34365906) and 21 putatively truncating, but heterozygous variants were present in the ∼60 000 individuals queried through the Exome Aggregation Consortium (ExAC) Browser. None of the three mutations reported here was in ExAC (though R→Q substitutions at both R219 and R748 are present).

The catalytic HECT domain of HACE1 is located at amino acids 572–909, with its active cysteine residue at position 876 (see ref. 8, in which HACE1 is referred to as hectH20). All three of the mutations described here, therefore, predict translation of a truncated HACE1 protein lacking part or all of the catalytic domain. Deletion of the HACE1 HECT domain, or mutation of the conserved HECT domain cysteine residue 876 (p.C876S), has previously been shown to cause loss of E3 ubiquitin ligase activity.^9^ ^10^ The truncated proteins encoded by all three mutant *HACE1* alleles can therefore confidently be assumed to be devoid of catalytic activity.

We attempted to demonstrate the presence of truncated protein in patient fibroblasts from family B. While no HACE1-specific signal was detected using a commercially available antibody against the N-terminal region of HACE1 (Origene), an antibody recognising the C-terminal HECT domain (Abcam) yielded a specific band of the correct size (∼102 kDa) in control fibroblasts. As predicted, this band was completely absent in cells from patients 6 and 7, confirming that mutant HACE1 is truncated and/or subject to nonsense-mediated mRNA decay (figure 1E).

Given the normal phenotype of multiple carriers within family A, and the absence of detectable HACE1 protein in fibroblasts in family B, it is also highly likely that these mutant alleles are null, rather than having any dominant negative function.

## Discussion

Here we report two families with eight affected children with ID and other overlapping clinical features, including muscular hypotonia, spasticity and ocular abnormalities. Autozygosity mapping and whole exome sequencing revealed three loss-of-function mutations in *HACE1.* In the consanguineous family, a homozygous nonsense mutation p.R219* was identified, while compound heterozygosity for another nonsense mutation p.R748* and a 20 bp insertion were identified in the German family. All three mutations are predicted to result in a truncated protein lacking the entire (p.R219*) or most of the catalytic HECT domain, and on that basis are assumed to be loss-of-function mutations. Western blot analysis of patient cell lines confirmed the absence of the C-terminal immunoreactive region; it remains possible that nonsense-mediated mRNA decay contributes further to the lack of detectable HACE1.

The finding of individuals who are apparently null for HACE1 function is unexpected, because *HACE1* has previously been postulated to be a tumour suppressor gene, inactivated in Wilms’ tumour and other cancers.^9^ ^11^ Support for this is derived partly from loss of expression and/or epigenetic changes in cancer cells; methylation changes resulting in reduced *HACE1* expression have also been associated with colon and gastric cancer.^12^ ^13^

However, in germline *Hace1*^–/–^ knockout mice, a predisposition for late-onset cancer and hypersensitivity to different carcinogenic factors was reported.^11^ In a child with bilateral, early-onset Wilms’ tumour, Slade *et al*^14^ identified a t(5;6)(q21;q21) translocation transecting HACE1. Among 421 additional Wilms’ tumour patients, one was found to be heterozygous for a truncating mutation (p.W364X), inherited from a healthy parent. Both of these presumed loss-of-function mutations were postulated to predispose to Wilms’ tumour.

While no truncating *HACE1* variants are identifiable in dbSNP and the 1000 genomes project, the current ExAC database includes 21 putatively truncating variants among 60 706 exomes. Also, none of the patients or heterozygous mutation carriers in either of our families was reported to have cancer. Although we cannot at present exclude a predisposition for late-onset tumours in the HACE1-deficient individuals, it therefore appears possible that the previously reported heterozygous mutations in cancer patients were incidental findings. Another putative tumour suppressor gene recently shown to be mutated in a neurodevelopmental syndrome is *WWOX.*^15^ ^16^ Again, in these studies patients homozygous for loss-of-function mutations, as well as carriers, showed no cancer predisposition.

*HACE1* (*KIAA1320*) is expressed in all regions of the brain and at lower levels in other tissues.^17^ As an E3 ubiquitin ligase, HACE1 recruits the E2 enzyme UBCH7 to ubiquitinate HACE1-specific target proteins for subsequent degradation by the 26S proteasome.^9^ One such target is the active form of Rac1,^18^ a member of the Rho GTPase subfamily that is involved in patterning cerebellar development by controlling cell morphogenesis, migration and foliation.^19^ In mice, dysregulation of Rac1 causes neurodevelopmental phenotypes that may be relevant to the human HACE1-deficiency phenotype; overexpression of constitutively active Rac1^20^ and knockouts of Rac1 regulators^21^ result in perturbations of cerebellar development manifesting with abnormal gait.

Rac1 is also involved in photoreceptor morphogenesis in *Drosophila* and mice.^22–24^ In particular, constitutively active Rac1 disrupts rod morphogenesis in mice, with defects in polarity and migration. It is therefore possible that the retinal dystrophy and macular hypoplasia that are present in four of our eight patients relate to a loss of Rac1 regulation by HACE1.

HACE1 also interacts with members of the Rab small GTPase subfamily. Rab1 appears to recruit HACE1 to the Golgi, where it plays a role in disassembly of the complex in mitosis.^10^ HACE1 is also reported to promote the recycling of the β^2^-adrenergic receptor (β^2^-AR) through a Rab11a-dependent mechanism.^25^

Besides its function as a E3 ubiquitin ligase, Zhao *et al*^26^ reported an E3-ligase independent interaction through which HACE1 represses the transcriptional activity of retinoic acid receptors RARα1, RARβ isoforms 1, 2 and 3; HACE1 thereby also represses the RAR-regulated genes CRABPII, RIG1 and RARβ2. The diverse regulatory roles of the RAR family imply several possible downstream actions of HACE1; for example, the CRABPII/RAR pathway mediates neuronal differentiation,^27^ while RARβ2 is involved in neuronal differentiation^28^ and regeneration and stimulation of neurite outgrowth.^29^

In summary, we described here eight HACE1-deficient individuals*.* Rather than the tumour predisposition phenotype that might have been predicted from earlier literature, all have a neurodevelopmental presentation. The patients show overlapping clinical features such as ID, verbal dyspraxia, muscular hypotonia, spasticity and ocular anomalies; however, the severity appears to be quite variable, particularly between family A and family B. Further molecular study of HACE1 in neurodevelopment, as well as identification of other HACE1-deficient subjects, should permit a better understanding of the physiological function of this gene and its genotype–phenotype relationships.

## Supplementary Material

Web figures

Web table

Web video
