# Supplementary material for: HACE1 deficiency causes an autosomal recessive neurodevelopmental syndrome
Source: J Med Genet. 2015 Sep 30;52(12):797–803. doi: 10.1136/jmedgenet-2015-103344 (PMC4717446; doi:10.1136/jmedgenet-2015-103344)
Supplement: Web figures [file jmedgenet-2015-103344-s1.pdf]

**Supplementary Figures:**

**Supplementary Figure 1**

Autozygous intervals in patients 1–5 (left to right), defined using Affymetrix SNP 6 data and displayed using AutoSNPa [5]. The sixth column is data from an unaffected individual. Homozygous genotypes are black, heterozygous yellow.

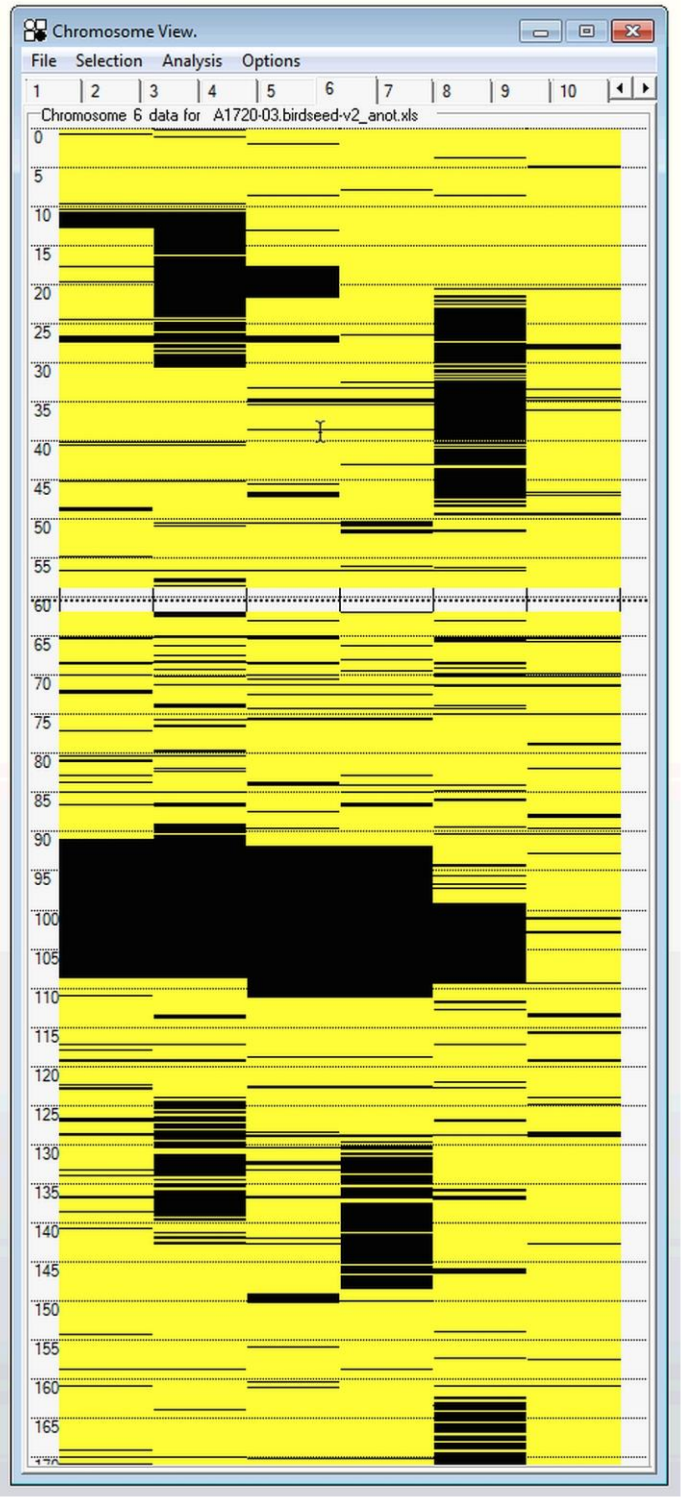

**Supplementary Figure 2**

Images of affected members of Family B.

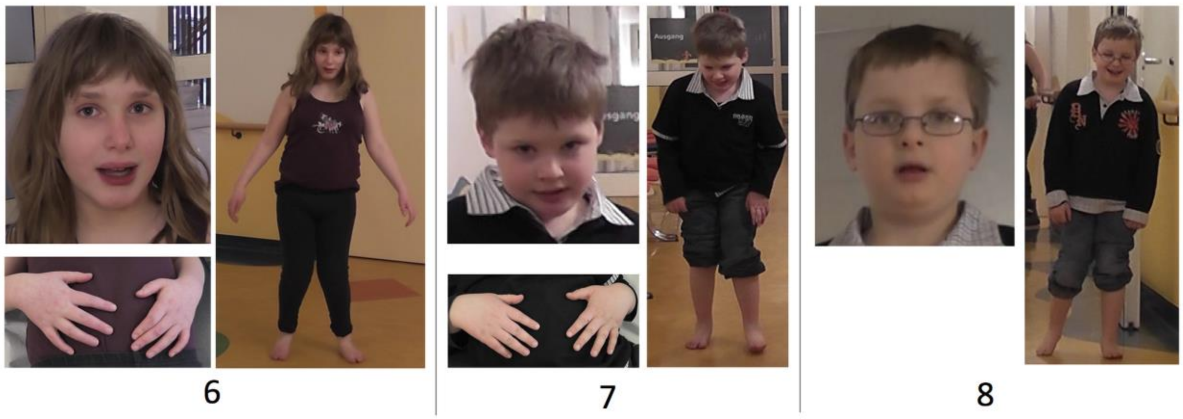


6 7 8
