# Supplementary material for: HACE1 deficiency causes an autosomal recessive neurodevelopmental syndrome
Source: J Med Genet. 2015 Sep 30;52(12):797–803. doi: 10.1136/jmedgenet-2015-103344 (PMC4717446; doi:10.1136/jmedgenet-2015-103344)
Supplement: Web table [file jmedgenet-2015-103344-s2.pdf]

GENE	HG19	TRANSCRIPT	EFFECT	VARIANT
LRRC7	chr1:70505469	uc001deq.3	missense	c.1571T>C; p.Leu524Ser
PHTF1	chr1:114253004	uc001edm.2	missense	c.412T>C; p.Met138Val
ENO4	chr10:118638829	uc021pzj.1	missense	c.565G>A; p.Ser522Asn
MYLK3	chr16:46781792	uc002eei.4	missense	c.314G>A; p.Ala105Val
ARRDC2	chr19:18120840	uc002nhu.3	missense	c.826G>A; p.Ala276Thr
OR5K2	chr3:98217047	uc011bgx	missense	c523A>G; p.Asn175Asp
C9	chr5:39341717	uc003jlv	missense	c.269G>A; p.Pro90Leu
CCDC152	chr5:42759312	uc003jmx	splice	NA
ZFYVE16	chr5:79733396	uc003kgs	missense	c.892A>G; p.Lys298Glu
KCNQ5	chr6:73904824	uc011dyk	missense	c.1736C>G, p.Ser579Cys
PHIP	chr6:79752720	uc003pir	missense	c.440G>A; p.Ala147Val
DOPEY1	chr6:83839024	uc010kbl	missense	c.2111G>A; p.Arg704Gln
CNR1	chr6:88854123	uc011dzt	missense	c.871C>G; p.Val291Leu
HACE1	chr6:105198317	uc003pqu	nonsense	c.2242G>A; p.Arg748*
HACE1	chr6:105219260	uc003pqu	frameshift	c.2019_2020ins TTTAGGTATTTTTAGGTATT; p.P.674Ffs*5
POLR3D	chr8:22106736	uc003xbl	missense	c.835C>G; p.Pro279Ala
CYHR1	chr8:145690273	uc003zcy	missense	c.12C>G; p. Lys4Asn
POLR1E	chr9:37486523-5	uc003zzz	indel	c.86_88delCCT
GCNT1	chr9:79117513	uc022bif	frameshift	c.215_216insG
ZNF484	chr9:95610353	uc004asv	missense	c.608A>G; p.Ile203Thr
FBP2	chr9:97349648	uc004auv.3	missense	c.274T>C; p.Thr92Ala

Supplementary Table S1

Exome data analysis of all three patients of family B; screen for shared homozygous or compound heterozygous non-synonymous variants. Known SNPs with “rs” numbers were excluded. *HACE1* mutations are shaded in grey.
